# Reduction en masse of inguinal hernia after self-reduction: a case report

**DOI:** 10.1186/s13256-021-02845-y

**Published:** 2021-05-04

**Authors:** Khosrow Najjari, Hossein zabihi Mahmoudabadi, Seyed Zeynab Seyedjavadeyn, Reza Hajebi

**Affiliations:** Department of Surgery, Sina Hospital, Tehran University of Medical Sciences, Imam Khomeini St., Tehran, Iran

**Keywords:** Hernia, Reduction, Incarceration

## Abstract

**Background:**

Reduction en mass (REM) is one of the rare complications of inguinal hernia reduction. Although REM can be detected on the basis of specific computed tomography (CT) scan findings, many radiologists are not familiar with its radiological appearance because of the scarcity of this complication, which may cause a delay in diagnosis.

**Case presentation:**

The patient reported in this article was a 50-year-old Persian man with a history of inguinal hernia, who had been referred with the periumbilical pain that radiated to the right lower quadrant and developed following hernia replacement by the patient himself. REM diagnosis was based on clinical examination and CT scan findings, and surgical treatment was performed by the Lichtenstein repair and mesh implantation.

**Conclusions:**

Although REM usually occurs after reduction with compression in the inguinal hernia, this unique case report highlighted the possibility of REM after self-reduction. Surgeons and radiologists should consider REM in patients with a history of inguinal hernia presenting with intestinal obstruction symptoms, even without any apparent signs of hernia in the physical examination.

## Background

Reduction en mass (REM) is one of the rare complications of inguinal hernia reduction inside the abdomen. REM is defined as the placement of the herniated sac inside the abdominal wall while the contents of the sac remain incarcerated or strangulated. Commonly, this sac stays in the preperitoneal space [1].

REM usually occurs after reduction with compression in the inguinal hernia. Although REM can be detected through specific computed tomography (CT) scan findings, many radiologists are not familiar with its radiological appearance because of the scarcity of this complication, which may delay the diagnosis [2]. Here we report a case of REM following hernia replacement by the patient himself.

### Case presentation

A 50-year-old Persian man was admitted with periumbilical pain that radiated to the right lower quadrant from 12 hours earlier. The patient had a history of right inguinal hernia 3 years ago. Previously, he was hospitalized twice because of incarceration and was a candidate for surgery, but he had not consented to surgery. The patient also had a history of ischemic heart disease and right hip arthroplasty. Regarding habitual history, he consumed opium orally.

**Fig. 1:**
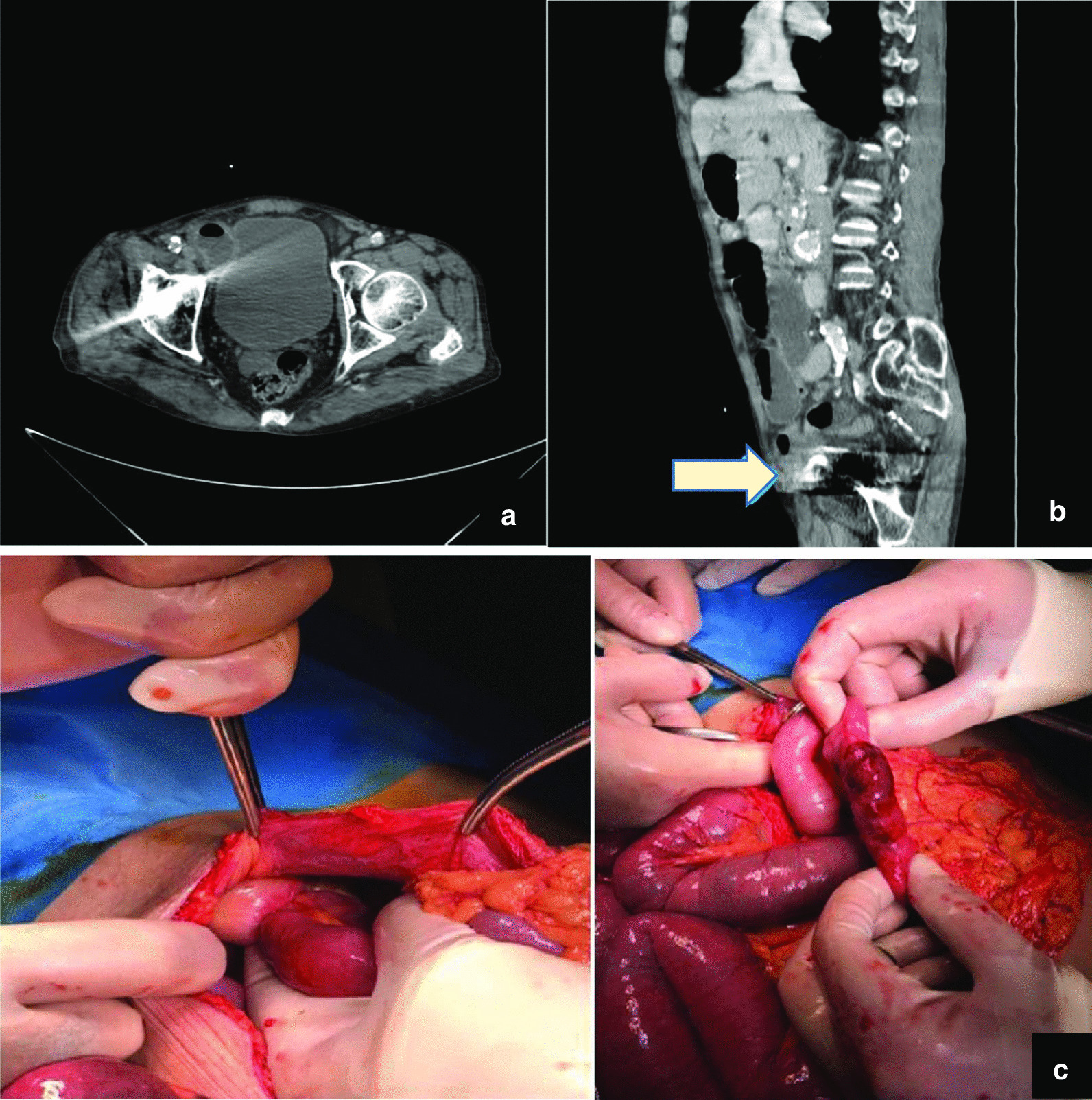
**a** Computed tomography scan of the abdomen and pelvis, **b** obstruction of small intestine and hernia sac and its contents inside the abdominal wall, **c** small intestinal loop with slight discoloration and adherence to the wall of the hernia sac inside the inner ring of the inguinal canal. The arrow in computed tomography indicates the hernia sac (under the fascia)

A day prior to admission and following inguinal hernia reduction by himself, the patient developed constant pain and a consequent inability to pass gas or stool. On physical examination, periumbilical and right lower quadrant tenderness (RLQ) was revealed. There was no acidosis or leukocytosis in the blood tests. Since there was no evidence of the previous hernia on the physical examination and the patient presented intestinal obstruction symptoms, including bilious vomiting 2–3 times, the medical team decided to obtain a computed tomography (CT) scan of the abdomen and pelvis (Fig. 1a). CT scan findings demonstrated the hernia sac and its contents inside the abdominal wall, as well as some evidence of obstruction in the small intestine (Fig. 1b).

The patient underwent laparotomy with a midline incision. Adherence to the wall of the hernia sac, and slight discoloration in a 5-cm-long intestinal loop approximately 50 cm before the ileocecal valve, which improved after about 10 minutes, were observed inside the inner ring of the inguinal canal (Fig. 1c). The intestinal loop was released from the sac without resection, the fascia and skin were then sutured, and the abdomen was closed. The inguinal hernia was incised classically in the next step, and a repair was conducted by the Lichtenstein method and mesh implantation. The patient was monitored for 5 days and finally discharged without any complications.

## Discussion

REM is one of the rare forms of acute intestinal obstruction encountered only by a limited number of surgeons and is unknown to many radiologists. Furthermore, REM is a rare complication in the inguinal hernia and can lead to intestinal obstruction, gangrene, and peritonitis in the case of delayed detection [1].

Imaging findings can be helpful in suspicious cases. A specific sign called the preperitoneal hernia sac sign can be found in REM imaging findings, which shows the incarcerated intestine located in the hernia sac inside the preperitoneal space [3].

According to Nason and Mixter [4], several criteria are required for REM to occur:

1. The hernia sac should have a narrow enough neck that makes it difficult for the intestine to exit the hernia sac.
2. The hernia sac should be mobile inside the hernial canal.
3. The hernia sac neck and the adjacent parietal peritoneum should be sufficiently mobile, thus allowing the sac to return into the peritoneal cavity without moving the intestinal loops.

There is usually a history of difficult reductions in the REM cases, among which the last reduction is usually the most difficult one and is followed either by the persistence of symptoms or the temporary improvement of them [2]. Moreover, in the physical examination, a painful mass can be felt in the proximal inguinal canal, above the inguinal ring, or in the lower quadrant on the reduced side.

Among the imaging modalities, magnetic resonance imaging (MRI) is reported to have the best performance for detecting groin hernias with 95% sensitivity and 96% specificity. However, because of the high cost of MRI, a CT scan with a sensitivity of 80% and a specificity of 65% and ultrasonography with a sensitivity of 86% and a specificity of 77% can be used instead to detect inguinal hernias [5, 6]. In our case, a CT scan was applied successfully.

Prompt surgical treatment is essential because any delay will exacerbate the symptoms and cause inevitable complications [1]. Interestingly, no masses or lesions were felt in our patient, and the patient only had RLQ tenderness. So far, limited REM cases have been reported after a hernia reduction in which the patient’s symptoms worsened, and the hernia sac had utterly entered the abdomen. However, in sporadic cases, the hernia was reduced by the patients themselves [7, 8].

## Conclusion

This unique case report highlights the importance of REM after self-reduction. Besides, this report stresses that a patient with a history of inguinal hernia and intestinal obstruction symptoms should be considered for REM, even without any apparent signs of hernia in the physical examination.

## Data Availability

All data generated or analyzed during this study are included in this published article.

## Abbreviations

CT: Computed tomography
MRI: Magnetic resonance imaging
REM: Reduction en mass
RLQ: Right lower quadrant
